# Baicalin Induces Apoptosis in SW620 Human Colorectal Carcinoma Cells *in Vitro* and Suppresses Tumor Growth *in Vivo*

**DOI:** 10.3390/molecules17043844

**Published:** 2012-03-29

**Authors:** Wen-Cheng Chen, Tsu-Hsiang Kuo, Yi-Shiuan Tzeng, Ying-Chieh Tsai

**Affiliations:** 1 Institute of Biochemistry and Molecular Biology, National Yang-Ming University, No.155, Sec.2, Linong Street, Taipei 112, Taiwan; Email: jistina1216@yahoo.com.tw (W.-C.C.); yishiuan1010@yahoo.com.tw (Y.-S.T.); 2 Graduate Institute of Life Sciences, National Defense Medical Center, Taipei 114, Taiwan; Email: tsuhsiang0923@yahoo.com.tw

**Keywords:** cancer, apoptosis, SW620

## Abstract

In the United States, colorectal cancer (CRC) is the second most frequent malignancy and the fourth most common cause of cancer death. Baicalin, a flavone derivative isolated and purified from the dry root of *Scutellaria*, was assessed for its antitumor effects in human SW620 CRC cells. Baicalin (200 μM) inhibited proliferation of SW620 cells. Baicalin (200 μM) increased activities of caspase-3, -8, and -9 in SW620 cells. Furthermore, flow cytometric analysis of baicalin-treated SW620 cells showed an increase in sub-G1 cells, and the dihydroethidium assay showed significant enhancement of intracellular peroxide production in baicalin-treated cells. Addition of *N*-acetylcysteine prevented most of the baicalin-induced apoptosis, which in turn mediated cytotoxicity in human SW620 cells. *In vivo*, baicalin (50 mg/kg/day, i.p.) treatment inhibited 55% of tumor growth in xenografted nude mice by 4 weeks, compared to that of the vehicle control (*p* < 0.05). Baicalin had no noteworthy influence on body weight. Thus, we suggest the development of baicalin as a potential leading antitumor agent in CRC.

## 1. Introduction

Colon cancer is diagnosed on approximately 1.2 million people worldwide and causes approximately 600,000 deaths annually [1,2]. Risk factors linked to colorectal carcinoma (CRC) reflect common lifestyle choices and include diet, exercise, and obesity. However, to state that it is primarily a lifestyle disease would be too much of a generalization [3]. Chances of survival for patients with late stage CRC are low, despite current treatments such as surgical excision, chemotherapy, and radiotherapy [4]. Thus, novel therapeutic agents that target additional mechanisms in CRC carcinogenesis are needed.

**Figure 1:**
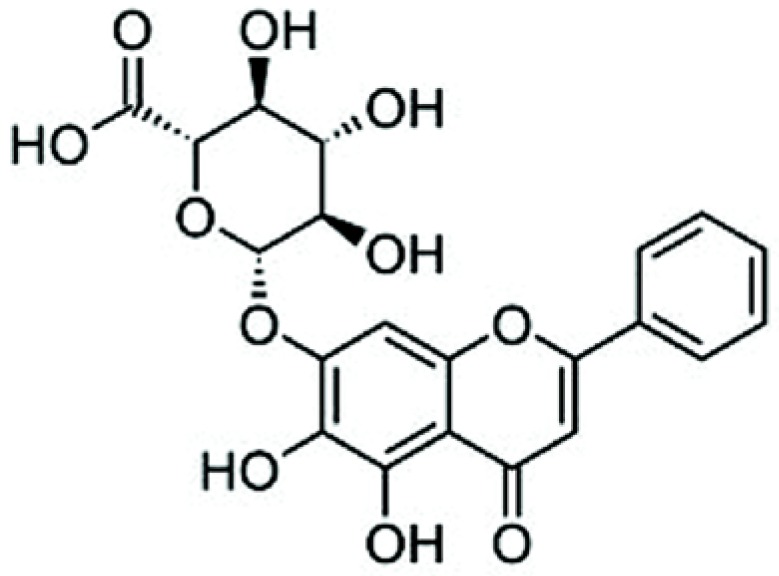
Chemical structure of baicalin.

*Scutellaria* is a remedial plant, whose dried roots are used in numerous Asian countries for various illnesses such as inflammatory diseases, allergy, diarrhea, hepatitis, and malignant tumors [5,6,7,8,9,10]. *Scutellaria* contains many flavonoids, which are usually found in the form of glucosides. It also includes amino acids, essential oils, and sterols [10]. Baicalin (CAS registry No. 21967-41-9; molecular weight for C_21_H_18_O_11_: 446.37; Figure 1) is found to be a chief active compound in *Scutellaria’s* anti-inflammatory and antitumor activity *in vitro* and *in vivo* [5,6,7,8,9,10]. Proliferation *in* *vitro* of human prostate cancer and promyeloleukemic and hepatocellular carcinoma cells is inhibited considerably by baicalin [10]. To date, baicalin-induced apoptosis in SW620 human colorectal carcinoma has not been described through any particular molecular mechanism.

Reactive oxygen species (ROS) are generally known to play a part in apoptosis, differentiation, and proliferation [11]. Excess ROS in the mitochondria appears to induce apoptosis [11]. Oxidative stress is a potentially cytotoxic result of excess ROS, that is, levels exceeding the local buffering capability such as in the mitochondria. Direct or indirect ROS action seems to be the main inducer of cytochrome c release from the mitochondria that triggers caspase activation [11]. The objective of this study was to assess whether baicalin inhibits growth of SW620 cells by inducing apoptosis. Thus, for this experiment, we examined the involvement of baicalin-induced oxidative response and apoptosis in antitumor activity against human SW620 CRC cells. 

## 2. Results

### 2.1. Apoptotic Activity on SW620 Cells

**Figure 2:**
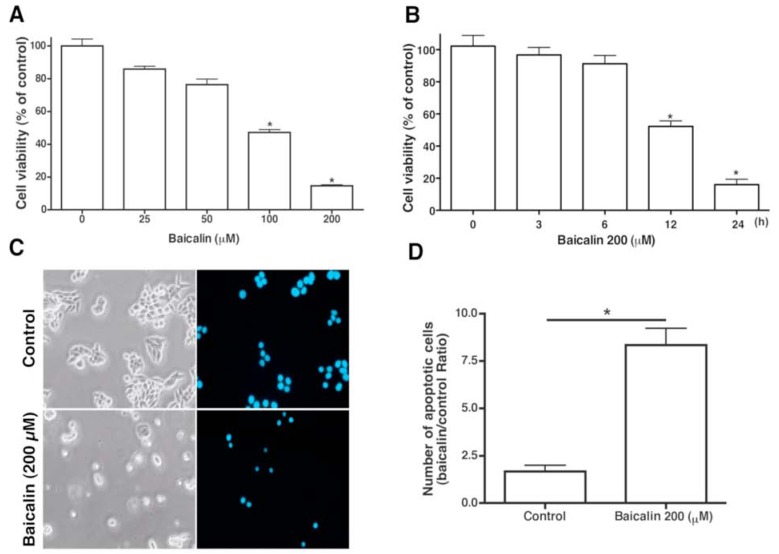
Effect of baicalin on human SW620 CRC cell viability, DNA fragmentation, and morphology. (**A**) Cell viability of SW620 cells treated with baicalin (25–200 µM) for 24 h. (**B**) Cell viability of SW620 cells treated with 200 µM baicalin for 3, 6, 12, and 24 h was measured by MTT assay. (**C**) DAPI staining for changes in nuclei. Treatment of SW620 cells with 200 µM baicalin or vehicle for 24 h with subsequent DAPI staining. Condensed and fragmented nuclei indicated presence of apoptotic cells. Magnification, ×200. (**D**) Fluorescence microscopy was performed to quantify the number of apoptotic cells. Three experiments were executed in triplicate. * *p* < 0.05, compared to the control group.

The effect of various concentrations of baicalin on the cell viability of SW620 cells was examined at 24 h by using MTT assays. A dose- and time-dependent loss of cell viability after exposure to baicalin was observed (Figure 2A,B). A noticeable loss of viability was detectable within 24 h of incubation at a baicalin concentration of 200 µm. Examination of the changes in SW620 cell morphology after exposure to 200 µm of baicalin for 24 h showed baicalin-induced cell toxicity; characteristics of apoptosis include formation of apoptotic body, cell shrinkage, rounding, and detachment (Figure 2C). Nuclear condensation, which is characteristic of apoptosis, was also observed with DAPI staining (Figure 2C). Baicalin-treated cells had a significantly higher proportion of apoptotic cells than untreated cells (Figure 2D).

### 2.2. Flow Cytometric Assessment of Baicalin-Induced Apoptosis

**Figure 3:**
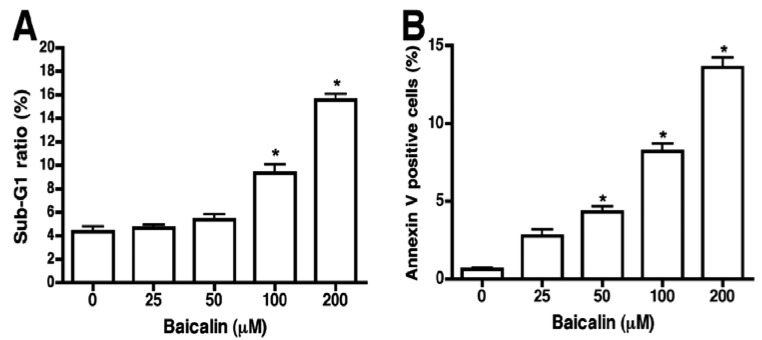
Quantification of sub-G1 cells in SW620 cells treated with baicalin. SW620 cells were cultured in the absence (control) or in the presence of baicalin at the indicated concentrations, stained with propidium iodide and Annexin V, and analyzed by flow cytometry. (**A**) Percentage of sub-G1 population of SW620 cells as measured by flow cytometry. (**B**) Flow cytometry with Annexin V antibody was used to analyze induction of apoptosis. The percentage of Annexin-V positive cells increased in conjunction with baicalin concentration in SW620 cells. * *p* < 0.05, compared to the control group.

Sub-G1 and Annexin V/PI staining were used to further characterize whether baicalin-induced cell death was mediated by apoptosis. Degradation and subsequent leakage of DNA from cells is a widely accepted hallmark of apoptotic cells [12], and cells with low DNA content (*i.e.*, hypoploid cells or sub-G1 cells) are also considered an indicator of apoptosis. PI staining of DNA indicates a lack of cell membrane integrity and can quantitate cellular DNA content. Distribution of nuclear DNA content was analyzed on flow cytometry to elucidate whether baicalin inhibition of SW620 cell growth was closely linked to apoptosis. Baicalin (200 µM) significantly increased the percentage of sub-G1 cells in SW620 cells to 14%, with a background of 4% in untreated cells (Figure 3A). Baicalin (25 to 200 µM) increased sub-G1 cells in a dose-dependent manner. Supporting these data, SW620 cells treated with baicalin exhibited a dose-dependent increase in Annexin V positive signal when detected by flow cytometry with an approximate 4-fold increase at a baicalin concentration of 200 μm, compared to untreated cells (Figure 3B). Overall, these experiments show that baicalin triggered apoptosis in SW620 cells.

### 2.3. Caspase Activation

**Figure 4:**
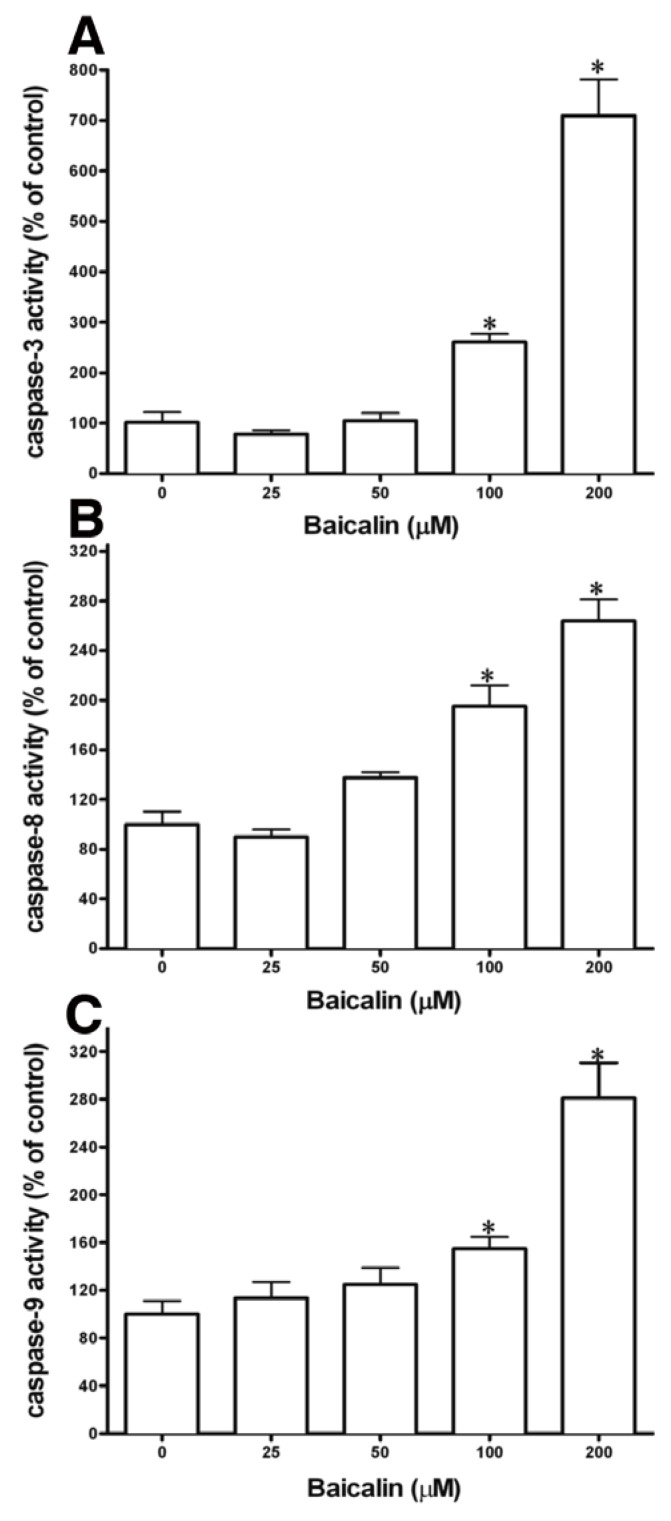
Baicalin’s effects on caspase-3, -8 and -9 activities. SW620 cells were treated with 200 µM baicalin for 48 h. Caspase activities of SW620 cells were determined with caspase activity detection kits. (**A**) Caspase-3, (**B**) caspase-8, and (**C**) caspase-9. Three experiments were performed in triplicate. * *p* < 0.05, compared to the control group. Reduce size of A–C in figures.

Caspase-8 and -9 respectively trigger the extrinsic and intrinsic cascades in the process of apoptosis. Different factors activate the pro-forms of the initiating caspases. Procaspase-8 is activated by membrane-associated proteins, and mitochondria-dependent activation is needed for procaspase-9 [13]. The roles of caspase-3, -8, and -9 in baicalin-induced cell apoptosis were explored by treating SW620 cells with 200 µm baicalin for 48 h and assessing the enzyme activity of caspase-3, -8, and -9 with colorimetric assay kits (Figure 4). Caspase-3 activation results in apoptosis, and baicalin treatment significantly augmented caspase-3 activity, compared to control cells (Figure 4A). Baicalin treatment also significantly increased caspase-9 activity (Figure 4C) and caspase-8 activation, compared to those in SW620 control cells (Figure 4B). Thus, induction of apoptosis through initiator caspases (caspase-8 and -9) and the effector caspase (caspase-3) was demonstrated by baicalin. The results show that the increased caspase activity by baicalin to induce apoptosis can inhibit SW620 cell growth.

### 2.4. Baicalin-Induced Generation of Intracellular ROS and Caspase-Dependent Apoptosis

**Figure 5:**
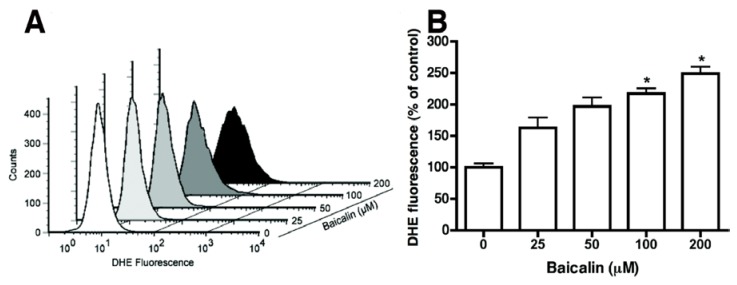
ROS production in SW620 cells. (**A**) Cells were treated with baicalin at the indicated concentrations for 24 h, and intracellular ROS levels were quantitated with FACS analysis. FACS profiles for DHE in untreated (vehicle) or baicalin-treated cells (10^4^ cells/sample) are representative of the 3 experiments. (**B**) Relative percentage of cells that showed detectable ROS production. Control ROS^+^ cells were set at 100. Three experiments were performed in triplicate. * *p* < 0.05, compared to the control group.

The process leading to baicalin-induced apoptosis was broken down by baicalin to elevate intracellular ROS levels, which were assessed with the cell-permeable, ROS-sensitive dye DHE [14]. Excess ROS activates DHE to become fluorescent, and ethidium intercalates into DNA [15]. Baicalin-induced production of oxidants in SW620 cells was monitored by the relative levels of DHE oxidation. Baicalin increased DHE fluorescence, indicating a higher ROS content that led to population shifts (Figure 5).

### 2.5. ROS Neutralization Conferred Resistance to Baicalin-Induced Apoptosis

**Figure 6:**
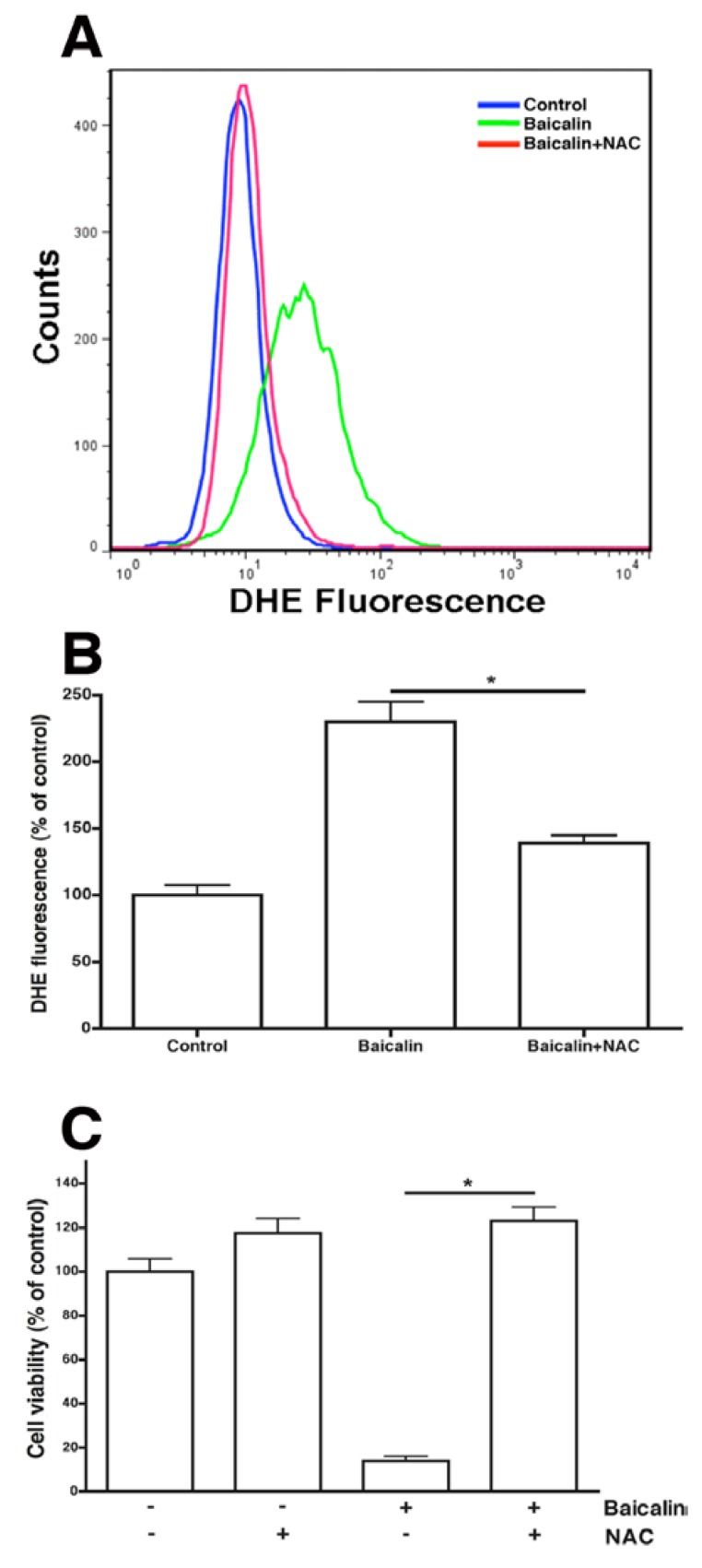
Effects of NAC and ROS production on cell viability. SW620 cells were incubated for 24 h with baicalin (200 µM) and ROS scavenger, NAC (2 mM), as indicated. (**A**) Intracellular ROS levels were quantitated with FACS analysis. FACS profiles for DHE in untreated (vehicle) or baicalin-treated cells (10^5^) were representative of the three experiments of three replicates. (**B**) The relative percentage of cells that showed detectable ROS production in the cell samples. Control ROS^+^ cells were set at 100. (**C**) Cells were incubated with or without baicalin (200 µM) in the presence or absence of ROS scavenger NAC (2 mM) for 24 h, and the proportion of surviving cells was measured with MTT assay. * *p* < 0.05, compared to the group treated with baicalin only.

The results show that baicalin-enhanced production of intracellular ROS coincided with baicalin-induced apoptosis. The roles of ROS in baicalin-induced apoptosis were examined by treating SW620 cells with baicalin in the presence or absence of *N*-acetylcysteine (NAC), a reactive oxygen intermediate scavenger. NAC neutralized most of the baicalin-induced ROS production (Figure 6A,B). In addition, NAC prevented baicalin-induced cell death (Figure 6C). These results indicate the importance of ROS targeting for baicalin-induced apoptosis. These results show that baicalin cytotoxicity involves cellular oxidants.

### 2.6. Baicalin Antitumor Activity in SW620 Human Colon Adenocarcinoma Cells *in Vivo*

**Figure 7:**
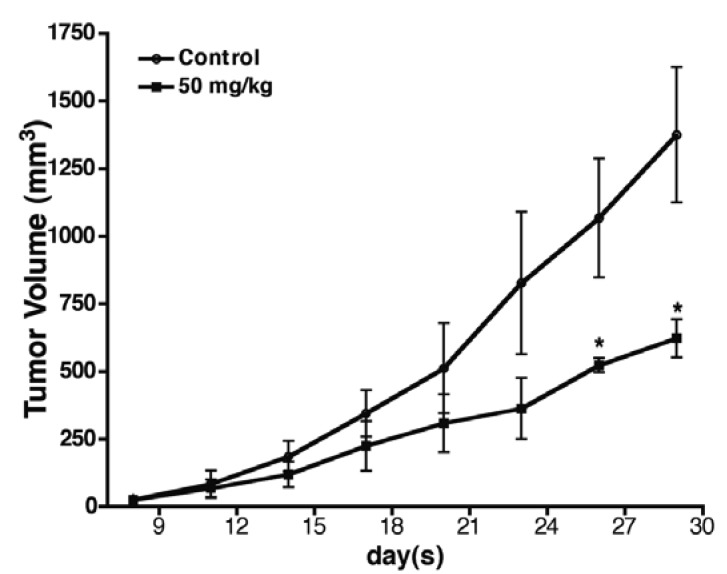
Effect of baicalin on the growth of colon cancer in the human SW620 CRC xenograft model. Xenograft experiments were performed as stated in the experimental section. Points, mean tumor volumes; bars, SE. Daily i.p. treatment of mice in the two groups: vehicle (n = 5) and 50 mg/kg baicalin (n = 5). * *p* < 0.05.

To assess the antitumor activity of baicalin treatment *in vivo*, SW620 human CRC xenografts [16] were implanted s.c. into nude mice. The mice were treated i.p. with baicalin (50 mg/kg) or a vehicle daily for 4 weeks. Baicalin (50 mg/kg) inhibited the growth of inoculated SW620 in mice. On Day 28 of treatment, baicalin had significantly inhibited tumor growth by 55% (*p* < 0.05) (Figure 7). In addition, no significant difference emerged in the average body weight of baicalin-treated mice, compared to that of the control mice (data not shown), indicating that mice receiving baicalin treatment do not show obvious toxicities.

## 3. Discussion

Most anticancer agents can be broadly grouped into blocking and suppressing mediators, which prevent tumor initiation or arrest the promotion and development of tumors, respectively [17,18]. Tumor-suppressing agents probably interfere with essential factors that control apoptosis, cell proliferation, or differentiation [19]. Numerous natural consumables and Chinese medicines are regarded as tumor-blocking or suppressing mediators [20]. Baicalin exhibits broad antitumor activity against human prostate cancer and promyeloleukemic and hepatocellular carcinoma cells [5,6,7,8,9,10]. Baicalin inducing apoptosis in malignant cells shows that baicalin can be a potentially significant element in chemoprevention.

ROS play an important role in certain apoptotic pathways [11]. In this study, baicalin increased a demonstrable level of intracellular ROS, detected with a DHE fluorescent probe (Figure 5). DHE was usually considered a probe for measuring ROS. Our results suggest that ROS may be involved in baicalin-induced apoptosis. This is in agreement with other studies that showed maintenance of JNK or ERK activity through the increase of ROS to cause cell apoptosis [21,22]. NF-κB and JNK activation may play opposing roles in the signaling pathways [23,24]. Moreover, transcription factors such as NF-κB activation by ROS have been associated with chromatin remodeling and proinflammatory-related gene expression [25]. Our results indicated that NAC nearly prevented or neutralized baicalin-induced ROS production and apoptosis (Figure 6). In previous articles, ROS production through Fas- and FADD-dependent pathways was indicated as DNA-damaging reagents that induce cell apoptosis. Effector caspases are activated when caspase-8 and -9 pathways combine in the death receptor and mitochondrial pathways [26]. We showed that baicalin exposure on SW620 cells caused time- and dose-dependent apoptosis through ROS production, which activated caspase-3, -8, and -9 (Figure 2–Figure 4). Regulatory proteins for cell proliferation such as c-Myc and Cdc25A are also related to the incitement or suppression of apoptosis [27,28], but the effects of baicalin on these factors are unknown in SW620 cells. Human SW620 CRC cells are p53 mutant cell lines, and the intact p53 gene is involved in stimulating DNA repair. Thus, further experiments are required to examine the effects of possible inactivation of the p53 gene on baicalin-induced apoptosis.

Certain phenolic compounds in phytochemicals have been shown to inhibit the metastasis of cancer, of which the flavonoids, plentiful in foods and numerous supplements, have been shown in increasingly more scientific reports to be extremely helpful and a natural hindrance in the infiltration and metastasis of cancer cells. For this study, the growth of SW620 cells was inhibited with baicalin. Remedial flavonoids such as apigenin, genistein, luteolin, and quercetin have been found to reduce mortality from some malignancies including breast, colon, and prostate cancers[29,30,31]. DHE oxidation level measurements revealed baicalin-induced oxidant production in SW620 cells by the action of baicalin. This caused a population shift because of the high ROS content (Figure 5). Previous reports have shown that reactive oxygen species (ROS) produced by apigenin, genistein, luteolin, and quercetin increase the death of cells through apoptosis[32,33,34]. Through this research, treatment of baicalin to SW620 cells intensified the activity of caspases in the intrinsic and extrinsic apoptosis pathways with the activation of caspase-3, -8, and -9. Activation of caspase-3 and -9 resulted in apoptosis caused by either apigenin or quercetin in leukemia and hepatoma cells, respectively [35]. Treatment of breast cancer cells to genistein led to apoptosis by activating caspase-12 and proapoptotic proteases [36]. The study also showed that caspase-8 of the extrinsic pathway in apoptosis was not engaged. Additional studies also show that quercetin in leukemia and colon cancer cells activated caspase-9 via the mitochondrial pathway; likewise, in rat hepatoma cell lines, caspase-9 can be activated by luteolin [37,38]. The treatment or application of flavonoids to cancer cells or other animal experiments suppresses tumor growth by reducing angiogenesis, cell-matrix adhesion, and EMT [39]. In addition, several protein activities such as upregulation in β1-integrin, cytokeratin-18, E-cadherin, and downregulation of CXCR4, CXCL12, EGF, EGFR, and MMP family proteins have also been reported in the effects of these biological processes [39]. The commonplace dietary flavonoids of the phenolic group have shown a wide range of activities *in vitro* and *in vivo* for the prevention of tumor growth and metastasis in many existing reports. However, whether baicalin in SW620 cells results in apoptosis through these pathways remains unclear. Therefore, this is a prospective aspect to clarify the downstream effects in apoptosis caused by baicalin in SW620 cells in the future.

## 4. Experimental

### 4.1. Cell Culture

SW620 cells were cultured in RPMI-1640 medium (Gibco) supplemented with 10% fetal calf serum (Gibco), 0.1 mM non-essential amino acids (NEAA), 2 mM glutamine, and 1% antibiotics (100 U/mL of penicillin and 100 μg/mL of streptomycin) in a humidified atmosphere containing 95% air and 5% CO_2_ at 37 °C.

### 4.2. Reagents

Baicalin from Sigma (St Louis, MO, USA) was dissolved in sterile dimethylsulfoxide (DMSO). Dihydroethidium (DHE), anti-β-actin, and *N*-acetylcysteine (NAC) were also obtained from Sigma.

### 4.3. Assessment of Cell Viability and Growth

Cell viability and growth were measured by MTT assay. Cells were briefly seeded at 2 × 10^4^ cells/mL for 18 h to 24 h before removal of the medium. Cells were incubated with baicalin at the indicated concentrations for 24 h and 48 h. The medium was replaced with an MTT solution (0.5 mg/mL) for 4 h. Formazan was dissolved with DMSO, and its concentration was measured spectrophotometrically at 595 nm. 

### 4.4. DAPI Staining

Fluorescence microscopy of 4,6-diamidino-2-phenylindole (DAPI)-staining was performed to detect morphological characteristics of apoptosis in the cell. An SW620 monolayer was fixed with 4% paraformaldehyde for 30 min at room temperature, permeabilized with 0.2% Triton X-100 in PBS (5 min, 3 times), and stained with DAPI (1 μg/mL) for 30 min. Apoptotic nuclei and intact cells (200–300 cells/sample) were scored at 200× magnification using a fluorescence microscope with a 340/380 nm excitation filter; the percentage was calculated.

### 4.5. Flow Cytometric Analysis

To perform propidium iodide (PI) staining, trypsinized cells were washed with cold PBS and fixed with 70% ethanol at −20 °C for approximately 1 h. After washing twice, fixed cells were treated with 1 mg/mL of RNase A and 0.5 mL of 0.5% Triton X-100/PBS at 37 °C for 30 min, and stained for 10 min with 0.5 mL of 50 mg/mL PI. Propidium-DNA complex was assessed using a FACSCalibur flow cytometer (BD Biosciences, San Jose, CA, USA). SW620 cells treated with baicalin for the specified time were examined by first resuspending the cells (2–3 × 10^6^ cells/mL) in an Annexin V binding buffer (10 mM HEPES/NaOH, pH 7.4, 140 mM NaCl, 2.5 mM CaCl_2_). Cell aliquots (100 µL/tube) were incubated with 5 μL of premixed Annexin V fluorescein isothiocyanate (FITC) (in dark for 15 min at room temperature). To distinguish necrotic cells, PI (5 µg/mL) was added and the samples (10^5^ cells) were analyzed using a FACSAnalyzer that quantified apoptotic cells (Annexin V^+^/PI^−^) (Becton-Dickinson). Three experiments with three replicates each are represented by the data shown.

### 4.6. Caspase Activity Assay

SW620 cells (approximately 5 × 10^5^ cells) were cultured in a 75 cm^2^ flask with 10 mL media for 12 h. After media removal, 10 mL test substances at the indicated concentrations or vehicle were incubated for indicated times. After rinsing, cells were harvested and assessed for caspase-3, -8, and -9 activities with colorimetric assay kits (BioVision, California, CA, USA), according to the manufacturer’s guidelines.

### 4.7. Measurement of Reactive Oxygen Species

DHE was used to indirectly determine the intracellular accumulation of ROS (O_2_^−^). Aliquots of cells (1 × 10^5^) individually received the oxidation-sensitive dye DHE (2 µM). After 15 min incubation, the concoction was diluted 10-fold with an ice-cold FACS buffer. Cells were washed and analyzed by FACS with the CellQuest software (Becton-Dickenson). For each sample, 10,000 cells were counted, and the percentages of the fluorescence intensity were compared with those of the control samples. 

### 4.8. Measurement of Tumor Volume in Nude Mice

Experiments were conducted according to the guidelines issued by the National Research Council’s Guide for the Care and Use of Laboratory Animals. The ethical approval number for the *in vivo* nude mice study is YM990702. Male BALB/c nude mice (35 to 40-d old, 18–22 g) were obtained from the National Animal Center (Taipei, Taiwan) and given standard laboratory food and water ad libitum in air-conditioned rooms with timed lighting conditions (12 h lighting/day). Human CRC SW620 cells were injected subcutaneously (s.c.) into the flanks or backs of nude mice and grown for 14 d. When the tumor had grown to 100 mm^3^ [16], the mice were randomly divided into two groups (n = 5). Each group was treated i.p. (vehicle control and 50 mg/kg baicalin) 7 times/week for 28 days. The size of the tumor was measured with vernier calipers once every 3 d in two perpendicular dimensions and then converted to tumor volume (TV) with the formula (ab^2^)/2, where a and b were the longer and shorter magnitudes, respectively. Body weights of the mice were measured daily for 4 weeks. At the end of testing, mice were sacrificed and weighed, and tumors were separated and weighed.

### 4.9. Statistical Analysis

Data of three independent experiments are presented as mean ± standard deviation (SD), and evaluated by one-way ANOVA. The results were considered significant when *p* < 0.05.

## 5. Conclusions

In these experiments, the natural flavonoid baicalin induced apoptosis in human SW620 CRC cells through ROS production, which activates caspase-3, -8, and -9. Baicalin has been demonstrated useful and should be further developed for enhanced treatment and control of human cancers as a novel anticancer agent. Most antitumor medicines include potent therapeutic effects but also serious side effects. Chinese herbs with greater efficacy and lower toxicity may be a possible source for novel drugs in tumor therapeutics. Baicalin had noticeable antitumor effects *in vivo* and showed no significant influence on body weight. In conclusion, baicalin has good potential as an antitumor drug for future treatment of CRC.
